# Individual Differences in Newborn Visual Attention Associate with Temperament and Behavioral Difficulties in Later Childhood

**DOI:** 10.1038/srep11264

**Published:** 2015-06-25

**Authors:** Kostas A. Papageorgiou, Teresa Farroni, Mark H. Johnson, Tim J. Smith, Angelica Ronald

**Affiliations:** 1 Faculty of Life Sciences and Computing, School of Psychology, London Metropolitan University, UK; 2 Centre for Brain and Cognitive Development, Department of Psychological Science, Birkbeck University of London, UK; 3 Dipartimento di Psicologia dello Svillupo e della Socializzzione, University of Padua, Padua, 35131, Italy

## Abstract

Recently it was shown that individual differences in attention style in infants are associated with childhood effortful control, surgency, and hyperactivity-inattention. Here we investigated whether effortful control, surgency and behavioral problems in childhood can be predicted even earlier, from individual differences in newborns’ average duration of gaze to stimuli. Eighty newborns participated in visual preference and habituation studies. Parents completed questionnaires at follow up (mean age = 7.5 years, SD = 1.0 year). Newborns’ average dwell time was negatively associated with childhood surgency (β = −.25, *R*^*2*^ = .04, *p* = .02) and total behavioral difficulties (β = −.28, *R*^*2*^ = .05, *p* = .04) but not with effortful control (β = .03, *R*^*2*^ = .001, *p* = .76). Individual differences in newborn visual attention significantly associated with individual variation in childhood surgency and behavioral problems, showing that some of the factors responsible for this variation are present at birth.

Recently it was shown that individual differences in infants’ attention at 4- to 10-months old significantly predicts behavior and temperament in childhood: the ability to hold fixations for a longer period of time as an infant was predictive of better effortful control, and less surgency and hyperactivity-inattention in later childhood^1^. An open question is to what degree these differences in infant attention, which predict later behavior, develop in the first months of life, perhaps as a result of caregiver and other environmental stimulation, and to what degree they are present at birth. The present study aimed to test this by investigating the degree to which individual differences in *newborns*’ visual attention is predictive of individual variation in effortful control, surgency and behavioral difficulties in childhood. Given that newborns have not yet been exposed to many environmental influences outside the womb, studying neonatal visual attention may facilitate our understanding of causal factors (e.g. prenatal factors and genetic factors) operating at birth to contribute to individual variation in attention, temperament and behavioral traits later in life. Investigating the causes of individual differences in visual attention as early as in the first hours after birth might inform early intervention practices that will aim to improve aspects of attention, a cornerstone of human cognition. Furthermore such investigation should in theory facilitate the early identification of individuals at risk for developing certain behavioral problems connected to attention difficulties, such as attention deficit hyperactivity disorder (ADHD).

## Parameters of newborn and infant attention

The most well studied attentional parameter in infancy (0–12 months) is the measure of *look duration*. The measure of look duration is usually measured using video cameras. It refers typically to participant’s longest time to habituate to a certain stimulus^2^. Shorter look duration is associated with better cognition, in theory because it shows that it takes a child less time to process and encode a new stimulus in working memory^2^. A different type of parameter that can be measured is the duration of individual eye fixations (typically the periods of relative oculomotor stability between saccadic eye movements) that take place during the ‘look’ at a stimulus. These individual fixations or saccades can be measured in several ways. Eye tracking offers the highest spatial and temporal resolution to capture *fixation duration*, the time between saccadic eye movements^3^.

How are look duration and fixation duration related ? Recent evidence derived from an eye tracking study suggests that mean fixation duration shows a trend towards a negative association with infants’ peak look duration–although this association did not reach statistical significance^4^. As such, the attentional style that is associated with better later cognition appears to be taking less time overall before habituating to a stimulus (i.e. having a *shorter peak look duration*) and having relatively *longer mean fixation duration*.

When studying newborns, collecting video coded data presents fewer practical challenges than eye tracking. As such, while it is difficult to obtain the mean fixation duration parameter (that requires eye tracking), it is possible from videos to derive more holistically coded measures of looking; for example the measure of average dwell time. Dwell time is best described as the duration that gaze remains upon individual stimuli^5^. In order to process a scene normally, adult viewers needed to foveate the scene for at least 150 milliseconds during each eye fixation^6^; in addition, the duration of fixations can be controlled on-line in an attempt to delay a saccade until encoding has completed^7^. As such, not all fixations succeed in delaying until encoding is finished. Taken together, these findings indicate that mean fixation duration and average dwell time are linked in adulthood.

A hypothesis is––based on the proposed link between fixation duration and dwell time in adults––that fixation duration and dwell time may be also linked in infancy. However, there is a developmental change in look duration in the context of a habituation paradigm over the first year of life, with different types of cognitive processing influencing looking durations at different ages^8^. As such, it is difficult to establish continuity in the relationship between average dwell time and mean fixation duration from infancy throughout childhood to adulthood. In addition, there are currently no studies that explored the degree to which average dwell time and mean fixation duration are linked across development. The present work has used dwell time as the best available proxy for fixation duration in a sample of newborns.

### Individual differences in infant attention

It has been suggested that between 3-to-12-months of age look duration exhibits high individual variation 2,4 and that it is the most stable measure of individual differences in visual attention across both short and long test-retest intervals^8^ and across different tasks^9^. The measure of look duration is used to classify participants into long-lookers (LL) or short-lookers (SL). Individuals are classified into LL or SL groups depending on whether their peak look duration is above or below the group’s median, respectively^2^. There is evidence to support the stability of individual differences in attentional style (whether someone will be classified as SL or LL) from infancy through toddlerhood. The scores of an individual on attentional measures in infancy have been found to correlate moderately with his/her scores on attentional and memory measures in toddlerhood (*R*^*2*^ = .18 and .12, respectively^10^,^11^).

Furthermore, individual differences in look duration have been found to correlate negatively with intelligence at 18-years of age; SL infants scored significantly higher than LL infants on IQ at 18-years of age (*R*^*2*^ = .09^12^). Finally, a recent longitudinal study has found that SL infants exhibited significantly higher executive function scores throughout early childhood (24–36-and-48-months of age) as compared to LL infants^13^. However it is noted that continuity between early visual attention and later executive control in Cuevas and Bell (2013) has been demonstrated in infants older than newborns. As such, a question remains whether continuity between early visual attention and later executive control could be demonstrated in a sample of newborns.

### Associations between infant attention and child temperament and behavior

A study explored the relationship between infant look duration and positive affect in infancy (indexed by the duration of 5-, 7-, and 9-month-old infant’s longest look^14^). The results demonstrated that LL participants scored higher than SL participants on positive affect at all ages in infancy^14^.

Recently we found that 4- to 10-month-olds’ mean fixation duration was positively associated with effortful control (*R*^*2*^ = .02) and negatively with surgency (*R*^*2*^ = .07) and hyperactivity-inattention (*R*^*2*^ = .06) in childhood. In the current study, we aimed to pursue this finding further by investigating whether these effortful control, surgency and behavioral problems in childhood can be predicted by attentional styles at an even earlier developmental stage, in newborns. In light of the evidence to date, we hypothesized that newborns’ average dwell time would be: (a) positively associated with childhood effortful control; (b) negatively associated with childhood surgency; (c) and negatively associated with childhood total behavioral difficulties.

The participant pool in this study comprised of 180 children, born between 2004 and 2012, who took part in visual preference and habituation studies carried out in the maternity ward of the Pediatric Unit of Monfalcone hospital in Italy (please see subsection titled “Visual studies” in the Method section for details). The common aim of these visual studies was to investigate newborns’ visual attention. The newborns were between 1-to-4 days of age (mean age in days = 2.20, SD = 1.20), when they took part in the studies. The final sample in the current analyses consisted of eighty participants (44 males, 36 females; mean age of the children in months when the parents competed the questionnaire = 90.00, SD = 11.80; please also see Method section for details on the study’s sample and procedure). For those eighty participants, the measure of total looking time on the screen was divided by the measure of total number of orientations, for each participant separately. This calculation produced a measure of the average dwell time: the time that the newborns spent looking at stimuli during the experiment per participant (please refer to supplementary material, section S2 for details). Questionnaire data was also collected from those eighty participants at follow up. The questionnaires assessed the temperament traits of effortful control and surgency (CBQ-sf; TMCQ) and behavioral difficulties (SDQ). Multiple linear regression was performed to test for significant associations (at *p* < .05 as each hypothesis was tested separately) between newborns’ average dwell time with effortful control, surgency, and total behavioral difficulties in childhood.

## Results

### Descriptive statistics

Descriptive statistics for average dwell time and for the scales of effortful control (z-score), surgency (z-score) and total behavioral difficulties for the whole sample are shown in Table 1. Table S1 in the Supplemental material available online presents the descriptive statistics for the CBQ and TMCQ scales of effortful control and surgency and the SDQ scale of total behavioral difficulties separately for the younger group of participants age 5 to 7 years and the older group of participants age 7 to 9.

### Sex differences

Females showed higher means than males in effortful control (F (1, 79) = .5.92, *p* = .01; see Table S2). No other statistically significant mean sex difference was observed.

### Mean group differences on visual measures between visual preference and visual habituation studies

There were statistically significant mean differences at *p* < .01 (corrected for multiple comparisons: p < .05/3 = .016; three is the number of comparisons between the two groups of participants for the three variables): Individuals who participated in visual preference studies exhibited longer total looking time (*F* (1, 79) = .41.92, *p* < .001 and greater number of orientations (*F* (1, 79) = .35.46, *p* < .001) than individuals who participated in the visual habituation studies. Importantly though, no statistically significant differences (at *p* < .01) between the two groups were observed for the measure of average dwell time at stimuli (*F* (1, 79) = .005, *p* = .94; see Table S3). Despite the fact that there were no differences in the measure of average dwell time at stimuli, the type of study that an individual took part in was treated as covariate in the regression analysis.

### Correlations between effortful control, surgency and total behavioral difficulties in childhood

Correlations between effortful control, surgency and total behavioral difficulties score are shown in Table 2, for all participants. Effortful control was correlated negatively at *p* < .01 (corrected for multiple comparisons) with surgency *(r* = −.37, *p* = .001) and total behavioral difficulties score (*r* = −.60, *p* < .001). As expected surgency was correlated positively with total behavioral difficulties and the result was significant (*r* = .28, *p* = .01).

### Multiple linear regression between newborns’ average dwell time at stimuli and effortful control, surgency and total behavioral difficulties in childhood

The results of the multiple linear regression for newborns’ average dwell time at stimuli (independent variable) with effortful control, surgency and total behavioral difficulties in later childhood (dependent variables) are shown in Table 3. Average dwell time was associated significantly (at *p* < .05) with surgency (*β* = −.25, *R*^*2*^ = .04, *p* = .02) and total behavioral difficulties score (*β* = −.28, *R*^*2*^ = .05, *p* = .04) but not with effortful control (*β* = .03, *R*^*2*^ = .001, *p* = .54). Scatter plots for the reported associations are shown in Figures S1, S2 and S3 in the Supplemental material for effortful control, surgency and total behavioral difficulties, respectively.

## Discussion

The aim of the current study was to explore the degree to which individual differences in newborns’ average dwell time associate with effortful control, surgency and total behavioral difficulties in childhood. As hypothesized, average dwell time was associated negatively with surgency and total behavioral difficulties in childhood. This is the first study to report an association between individual differences in attention in the first days after birth with temperament and behavior in childhood (5–9 years). As such, the results of the current study bridge the existing gap in the literature demonstrating that a measure of visual attention at birth is significantly associated with some behaviors in childhood.

The significant associations were of moderate magnitude, with the proportion of variance explained by newborns’ average dwell time at stimuli being 4% and 5% for surgency and total behavioral difficulties in childhood, respectively. Previous research reported that mean fixation duration in later infancy (4–10 months of age) explained 7% of the variation in surgency in early childhood (18–54 months of age) and that this association becomes stronger as the age of the infant increases^1^. The slightly lower proportion of variation in surgency that is explained by newborns’ average dwell time reported here could be explained either by the fact that predictor and dependent variable were further apart in developmental time as compared to previous research on older infants^1^; or the differences in the dwell time measure compared to the eye tracking-derived fixation duration previously used.

The non-significant association between newborn average dwell time and child effortful control could be explained from the fact that the association between infant attention and effortful control and in Papageorgiou et al. (2014) showed the trend of being the weakest association of the significant findings. In light of the smaller sample size in the present work, the negative result could be explained by lack of the power to detect a more modest association between newborn attention and effortful control.

Studying individual differences in newborns’ attention constitutes a window into the developmental mechanisms that contribute to individual differences in attentional and behavioral control throughout the lifespan. For example, attentional and behavioral control in childhood depends mainly upon overlapping cortical brain systems 15. Specifically, the executive attention system consists of at least two anatomically distinct brain systems, the cingulo-opercular network and the fronto-parietal network, that together form in large part the control system network of the brain and hold an important role in self-regulation. This proposal is supported by neuroimaging studies showing that brain areas that form part of the the cingulo-opercular executive attention network–like the anterior cingulate cortex–exhibit higher activation in tasks that include cognitive conflict, such as the color-word Stroop task^15^. However at birth (and prior to 3-months of age) attentional control depends upon subcortical brain structures^16^. The reported associations between newborns’ attention with some domains of temperament and behavior in childhood suggests that some of the underlying factors that may contribute to individual differences in the efficiency of young children to control their attention and behavior are present as early as at birth; that is, before the cortical systems that underlie behavioral and attentional control start to influence behavior.

These findings should be considered in light of some limitations. The measure of newborns’ attention was not derived from eye tracking data, which has much higher spatial (~1° of visual angle) and temporal resolution (typically between 50–300 Hz) in comparison to video coding (typically around 25Hz). For example, long dwells could be made up of a small number of long fixations or a large number of short fixations^7^. The significant associations reported in this study support the former possibility but some of the variance in the dwells might be due to the latter. As such, future research could use simultaneous eye tracking and video coding to estimate the correlation between average dwell time with fixation duration assessed using eye tracking and to attempt to replicate the findings of this study. However the video coded data used in this study were reliable as the Cohen’s Kappa inter-rater reliability was above .80 across all studies.

A second limitation of this work is that the measure of average dwell time was only hypothesised to be a proxy for mean fixation duration in infancy. It has been argued that there are at least three critical postnatal periods of attentional development in infancy^17^: the first involves the period from birth to 2–, 3–months of age, when the development of alert state takes place^17^. The second involves the period from 3 to about 6 months, when the orienting system emerges^17^. The third refers to the period from 6 months to 12 months, when executive attention is starting to practice control^17^. As such, looking measures may represent different underlying constructs at different points during the first year of postnatal life and later in development. For example, individual differences in newborn average dwell time may be more closely tied to the development of the alert state rather than to the development of the orienting or executive attention system. The lack of findings on the relationship between average dwell time and mean fixation duration across development and the fact that the current study did not take into account specific developmental stages of attention in infancy do not allow to state with certainty the degree to which the two measures are linked.

Another limitation of the study was the reliance on parent report of children’s behavior and temperament. While parents are typically most familiar with their children’s behavior, all types of raters include some bias. For example, using parent report measures of children behavior does not allow to disentangle whether the reported correlations represent continuity of an underlying endogenous attribute of the child; or whether the child’s early behavior feed into the development of adults’ perception of the child, which is then reflected in the parent report measures. Future research should consider collecting data from multiple raters or employing additional objective measurements of behavior.

In terms of how individual differences in children’s behavior and temperament are influenced, these data show that part of the variance in surgency and general behavior problems is explained by factors already present on the first or second day of life. As such, the origins of individual differences in surgency and general behavior problems are not likely to be wholly due to the postnatal environment. These causal factors could be genetic or stemming from the prenatal environment. For example, twin heritability estimates revealed that as much as 41% of the variation in surgency and 82% of the variation in behavioural problems like hyperactivity-inattention in childhood can be explained by genetic factors^18^,^19^.

Genetic research could investigate genetic variation that contributes to both individual differences in attention at birth and to individual differences in attentional and behavioral control in childhood. Longitudinal studies could investigate prenatal factors (e.g. prenatal maternal stress) that may contribute to individual differences in attention at birth and in temperament and behavior in childhood. Finally, neuroimaging could be used to explore individual differences in the development of the brain systems upon which newborns’ and infants’ attention is based^20^.

## Method

### Sample and procedure

The participant pool comprised of 180 children, born between 2004 and 2012, who took part in visual preference and habituation studies carried out in the maternity ward of the Pediatric Unit of Monfalcone hospital in Italy. The common aim of these visual studies was to investigate newborns’ visual attention. The newborns were between 1-to-4 days of age (mean age in days = 2.20, SD = 1.20), when they took part in the studies. Findings of the studies from which the visual data used in the current study were retrieved are presented in details elsewhere^21^,^22^,^23^,^24^.

The families were invited to participate in the follow up study by telephone and post between August 2013 and October 2013. One hundred and thirty one participants accepted the invitation (response rate = 73.0%). Following the acceptance of the invitation, they were given the option to either visit the lab in Monfalcone hospital or for our team to visit them at their homes to collect the questionnaire data. All participants returned fully completed questionnaires and signed consent forms. Twenty-nine participants were younger than 36-months of age. Those participants were excluded from the analysis because the focus of the study was to explore the relationship between newborns’ attention with temperament and behavior in children, as such participants had to be age 36 months or older to be included. There was missing visual data (those data could not be retrieved from the datasets) for twenty-two (of the remaining one hundred and two) participants. The final sample in the analyses thus consisted of eighty participants (44 males, 36 females; mean age of the children in months when the parents competed the questionnaire = 90.00, SD = 11.80). The majority of the participants (93.75%) were Italians and residents of Gorizia province; 5 families (6.25%) originated from Slovenia but they were also residents of Gorizia province and they could speak and write Italian.

The project was granted ethical approval by the Department of Psychological Sciences, Birkbeck University of London’s departmental ethics committee and the ethics committee of the University of Padua in Italy.

The methods were carried out in “accordance” with the approved guidelines; Informed consent was obtained from the parents of all children that took part in the current study.

### Visual studies

The visual studies had several important parameters in common: the stimuli that were used in the studies were always faces (human faces and schematic face-like configurations) with two stimuli presented at the same time side-by-side on the screen; all the experiments were run in the maternity ward of the Pediatric Unit of Monfalcone hospital in Italy; the equipment that was used to conduct the experiments was the same across all the visual studies; the analytic protocol for coding the visual data was the same across all studies; each individual that took part in the current study participated in only one of the experimental studies; all participants were born full-term, they were not older than 4-days of age and had an Apgar score of above 8 at five minutes after birth.

The studies followed either the visual preference (49% of the studies from which our data come from) or the visual habituation procedure (51% of the studies from which our data come from). A brief description of these procedures is given in Section S1 of the supplementary material. A description of the visual studies apparatus and stimuli is given in Section S2 of the supplementary material.

### Measures

#### Visual measures

The measure of total looking time on the screen was divided by the measure of total number of orientations for each participant separately. This calculation produced a measure of the average dwell time: the time spent looking at stimuli during the experiment per participant (please refer to supplementary material, section S2 for details). Number of orientations was coded in an identical way for both the visual habituation and visual preference studies. Specifically, the coders recorded, separately for each stimulus, how many times the newborn fixated on the stimuli (i.e. total number of orientations to the stimuli). Average dwell time did not differ significantly as a function of whether the newborn took part in a visual preference or visual habituation study (please refer to *Result* section below). Nevertheless, the particular experiment that an individual took part in the visual study was treated as a covariate in the analysis.

#### Questionnaires

Eight subscales of the short form of the Italian version of the *Childhood Behaviour Questionnaire* parent report (CBQ-sf; translated by Giada Matricardi^25^) that have been shown to load onto two factors, namely effortful control and surgency, were employed to assess temperament in 36–84-month-old children. Effortful control is the temperament trait of one’s ability to regulate his or her emotions and to inhibit a dominant response in order to activate a subdominant response^26^. Effortful control has been found to correlate negatively with impulsivity^27^ and hyperactivity^28^ and to differentiate reliably, typically developing children from children with attention-deficit/hyperactivity disorder (ADHD), with the latter scoring significantly lower than the former on measures of effortful control^29^. Surgency is the trait aspect of temperament in which a person tends toward high levels of extraversion, motor activity, and impulsivity, and it has been found to correlate with aggression and externalizing behavior problems in early childhood^30^. The scores on the Italian short form version of the CBQ scale of effortful control represented the average score of the CBQ subscales of inhibitory control (12 items), attentional focusing (12 items), low-intensity pleasure (13 items) and perceptual sensitivity (12 items). The scores on the Italian short form version of the CBQ scale of surgency represented the average score of the CBQ subscales of activity level (7 items), high-intensity pleasure (6 items), impulsivity (4 items) and shyness (6 items reversed)). The rater reported the frequency of a particular behavior (example question for effortful control: “*Your child prepares for trips and outings by planning things s/he will need; Si prepara per gite o uscite pianificando le cose di cui avrà bisogno”.* Example question for surgency: “*Your child likes to play so wild and recklessly that he/she might get hurt; Gli/Le piace giocare in modo così vivace e spericolato che si potrebbe far male*”, on a seven-point scale (ranging from “never/assolutamente falso” to “always/assolutamente vero”). The Italian version of the CBQ short form scales of effortful control and surgency showed excellent internal consistency (Cronbach’s alphas = .75 and .91, respectively) in the sample used in this study.

The equivalent eight subscales of the Italian version of the *Temperament in Middle Childhood Questionnaire* parent report (TMCQ; translated by Lavinia Barone^31^ were employed to assess effortful control and surgency in 84–120 month old children. The scores on the Italian version of the TMCQ scales of effortful control and surgency represented the average score of the same subscales as for the Italian version of the CBQ short form. The rater reported the frequency of a particular behavior (example question for effortful control: “*Your child can stop himself/herself when s/he is told to; Si ferma quando gli/le viene detto di farlo.* Example question for surgency: “*Your child likes rough and rowdy games; Gli/le piacciono i giochi scalmanati e chiassosi”)*, on a five-point scale (ranging from “almost always untrue; quasi sempre falso” to “almost always true; quasi sempre vero”). The TMCQ scales of effortful control and surgency showed excellent internal consistency (Cronbach’s alphas = .90 and .89, respectively) in the sample used in this study.

To assess behavioral difficulties, parent report of the total behavioral difficulties score of the Italian version of the Strengths and Difficulties Questionnaire (SDQ; translated by Andrea De Giacomo, Paola Dazzan and Loreta Bernardi^32^ was employed. The SDQ total behavioral difficulties scale represents the sum score of the scores on four scales, namely: Hyperactivity-inattention, conduct problems, emotional symptoms and peer problems. The rater reported on the frequency of a particular behavior (e.g. “*Restless, runs about or jumps up and down, doesn’t keep still; Irrequieto, iperattivo, incapace di stare fermo per molto tempo* ”) on a three-point scale (“*not true (non vero)”; “sometimes true (parzialmente vero)”; “certainly true (assolutamene vero)*”). The SDQ is a reliable and valid measure of total behavioral difficulties of children age 3 to 16 year olds^32^. The SDQ total behavioral difficulties score showed excellent internal consistency (Cronbach’s alpha = .80) in the sample used in this study.

### Statistical Analyses

#### Descriptive statistics

Average dwell time and the questionnaire data were explored using descriptive statistics in SPSS version 18.0. Due to skewness of the data, Van der Waerden’s transformation^33^ was used to normalize the data before further statistical analyses were undertaken. Levene’s test was used to test the assumption of equality of variances between males and females and Analysis of Variance (ANOVA) was performed to test for significant mean sex differences (at *p* < .01 corrected for multiple comparisons: p < .05/3 = .016; three represents the number of comparisons that were made between males and females). Similar analysis was performed to test for significant mean differences on average dwell time between the group of participants that took part in visual preference studies and those that took part in visual habituation studies.

#### Correlations

Partial correlations were performed to test for significant correlations (corrected for multiple comparisons at p < .05/3 = .016; three represents the number of correlations that were performed) between the questionnaire scales of effortful control, surgency and total behavioral difficulties. Sex and age of the child when the parents completed the questionnaires were used as covariates. In addition, whether the CBQ or TMCQ questionnaires were used was included as a covariate in the analysis. The two age groups were merged to increase the statistical power to detect significant associations; given that the parents rated the frequency of a particular behavior on a seven point scale on the CBQ and on a five point scale on the TMCQ, the scales of effortful control and surgency were z-scored for the two groups separately before merging the datasets.

#### Regressions

Multiple linear regression was performed to test for significant associations (at *p* < .05 as each hypothesis was tested separately) between newborns’ average dwell time with effortful control, surgency and total behavioral difficulties in childhood. The effects of age when the child took part in the visual study and the age of the child when the parents completed the questionnaire, the type of questionnaire that was completed by the parents (CBQ or TMCQ) and newborns’ total time to complete the visual experiment were treated as covariates in the regression analysis. Finally, the particular study (specifically the particular experiment within each study) that each newborn took part in was treated as covariate in the regression analysis.

## Additional Information

**How to cite this article**: Papageorgiou, K. A. *et al.* Individual Differences in Newborn Visual Attention Associate with Temperament and Behavioral Difficulties in Later Childhood. *Sci. Rep.* **5**, 11264; doi: 10.1038/srep11264 (2015).

## Supplementary Material

Supplementary Information

## Figures and Tables

**Table 1:** Descriptive statistics for average dwell time, effortful control, surgency and SDQ total behavioral difficulties for all participants.

	N = 80 (5–9 years of age)			
	Average Dwell Time (in ms)	Effortful Control (z-score)	Surgency (z-score)	SDQ Total Behavioral Difficulties Score
N	80	80	80	80
Mean	3,808	.00	.00	7.60
SD	1,963	.99	.99	5.46
Median	3,319	−.02	.00	7.00
Mode	559	−.01	−2.23	2.00
Minimum	559	−2.75	−2.23	.00
Maximum	12,389	2.31	2.47	27.00
Kurtosis	4.16	−.17	.20	1.58
Skewness	1.60	−.03	.16	1.09

**Table 2:** Correlations between effortful control, surgency and total behavioral difficulties in childhood.

	N = 80 (6–9 years of age)		
	Effortful Control	Surgency	Total Behavioral Difficulties
Effortful Control	1.00	−.37**	−.60**
Surgency	−.37**	1.00	.28**
Total Behavioral Difficulties	−.60**	.28**	1.00

^**^p < 0.01, * p < 0.05.

**Table 3:** Linear regressions between newborns’ average dwell time and effortful control, surgency and total behavioral difficulties score in childhood.

N = 80							
Independent Variable: Average Dwell Time							
Dependent Variable	B	β	t	95% CI for β Lower Bound	95% CI for β Upper Bound	R^2^	p-value
Effortful Control	.03	.03	.30	−.17	.23	.001	.54
Surgency	−.25	−.25	−2.24	−.49	−.02	.04	.02
Total Behavioral Difficulties	−.28	−.28	−2.09	−.55	−.01	.05	.04

Note: The “B” and “β” refer to the unstandardized and standardized regression coefficients respectively followed by the “t-statistic”. The “R^2^” represents the variance explained by the independent variable on the dependent variables after regressing out the effect of the covariates; finally, the p-value represents the value of statistical significance of the effect of the independent variable on the dependent variables, while keeping constant the effect of the covariates.
