# Supplementary material for: Individual Differences in Newborn Visual Attention Associate with Temperament and Behavioral Difficulties in Later Childhood
Source: Sci Rep. 2015 Jun 25;5:11264. doi: 10.1038/srep11264 (PMC4480143; doi:10.1038/srep11264)
Supplement: Supplementary Information [file srep11264-s1.pdf]

Individual Differences in Newborn Visual Attention Associate with Temperament and Behavioral Difficulties in Later Childhood

Kostas A. Papageorgiou*, Teresa Farroni, Mark H. Johnson, Tim J. Smith and Angelica Ronald

Faculty of Life Sciences and Computing, School of Psychology, London Metropolitan University, UK

Centre for Brain and Cognitive Development, Department of Psychological Science, Birkbeck University of London, UK

Dipartimento di Psicologia dello Svillupo e della Socializzzione, University of Padua, Padua, 35131, Italy

Author Note

Kostas A. Papageorgiou, Faculty of Life Sciences and Computing, London Metropolitan University; Teresa Farroni, Dipartimento di Psicologia dello Svillupo e della Socializzzione, University of Padua; Mark H. Johnson, Centre for Brain and Cognitive Development, Department of Psychological Science, Birkbeck University of London; Tim J. Smith, Centre for Brain and Cognitive Development, Department of Psychological Science, Birkbeck University of London; Angelica Ronald, Centre for Brain and Cognitive Development, Department of Psychological Science, Birkbeck University of London

This research was funded by grants to AR from the Royal Society and the Waterloo Foundation as well as the EC Marie Curie Initial Training Networks; the newborns experiments were supported by grants to TF from Welcome Trust (073985/Z/03/Z). MHJ is supported by the UK Medical Research Council. KAP is supported by the EC Marie Curie Initial Training Networks.

Correspondence concerning this article should be addressed to: Kostas A. Papageorgiou, Faculty of Life Sciences and Computing, London Metropolitan UniversityN7 8DB, UK; e-mail: k.papageorgiou@londonmet.ac.uk; phone: +44 (0) 2071332570

Method

Section S1. Description of visual preference and habituation procedure

Visual preference. In the visual preference studies (49% of the studies from which our data come from), the newborns sat on the experimenter's lap, 35cm distant from a translucent screen. The newborn holder was not actively involved in the experiment. As soon as the newborns started looking at the centre of the screen, the experimenter, who was watching the newborn's eyes via a video monitor system, initiated a trial and presented the stimuli on the screen. In accordance with the infant-control procedure, the stimuli remained on display for as long as the newborn looked at one or more of them (Slater, Morison, Town, & Rose, 1985). Newborns were presented with two trials in which the position of the stimuli was reversed. The initial side of the two stimuli, left or right was counterbalanced across the subjects. When they shifted their gaze from the display for more than 10 seconds, the experimenter turned off the stimuli. Two coders, who were blind to the location of the stimuli, analysed videotapes of the newborns' eye movements throughout the experiment. The coders recorded, separately for each stimulus and each trial, the number of orientations (how many times the newborn fixated on the stimuli) and the total fixation time at each stimulus. To help establish the position of the newborns' fixation, the coders could see the corneal reflection of the stimulus, but they were blind to the type of stimuli at display. The Cohen's kappa inter-rater reliability for 10% of the data was calculated for both the number of orientations and the total dwell time at stimuli. The Cohen's Kappa was above .80 for both measures across all the visual studies (Rigato, Johnson, Faraguna and Farroni, 2011; Farroni, Menon, Rigato, Johnson, 2007; Farroni, Menon, Johnson, 2006; Farroni, Johnson, Menon, Zulian, Faraguna and Csibra, 2005).

Visual habituation. In the visual habituation studies (51% of the studies from which our data come from), the newborns sat on the experimenter's lap, 35cm distant from a translucent screen. The experiments were carried out using a visual habituation technique with infant control procedure (Slater, Earle, Morison, & Rose, 1985). The infant was judged to have habituated when, from the fourth fixation

on, the sum of any three consecutive fixations was 50% or less than the total of the first three fixations. When the habituation criterion was reached, the stimulus was automatically turned off and a preference test phase started. The initial side of the two stimuli, left or right was counterbalanced across subjects. In accordance with the infant-control procedure the coding of the visual measures (total fixation time and number of orientations) in the habituation studies was performed online (Slater, et al., 1985). The beginning and the end of a trial were dependent upon the newborns' time to reach the habituation criterion. During the post-habituation phase two identical stimuli were presented. When the habituation criterion was reached, the stimulus was automatically turned off and a preference test phase started. Once the newborns looked at the centre of the screen, the experimenters pressed a button to indicate the start of the trial. The habituation was followed by a preference test (post-habituation) in which a preference could be expressed between the familiar stimulus and a novel one. The two test stimuli were shown in both left and right positions, the positions being reversed from the first to the second presentation. Each presentation lasted at least 20 seconds. A video camera recorded the newborns' eye movement to monitor their looking behavior and to allow off-line coding of their fixations. Subsequently, two coders, who were unaware of the stimuli presented, analyzed videotapes of the baby's eye movements throughout the trials. The coders recorded, separately for each stimulus and each trial, the number of orientations ((how many times the newborn fixated on the stimuli) and the total fixation time to stimuli. The experimenter coded an orientation when newborns were fixating at one of the two stimuli without going away for more than 2 seconds. The Cohen's Kappa was above .80 for both orientation and fixation time across all the habituation studies (Rigato, Johnson, Faraguna and Farroni, 2011; Farroni, Menon, Rigato, Johnson, 2007; Farroni, Menon, Johnson, 2006; Farroni, Johnson, Menon, Zulian, Faraguna and Csibra, 2005).

Section S2. Description of Visual Studies Apparatus and Stimuli

The aim of the studies was to investigate newborns' attention. The newborns were between 1-to-4 days of age (mean age in days = 2.20, SD = 1.20), when they took part in the visual studies. Each individual that took part in the current study participated in only one of the experiments presented below. The studies are presented in chronological order from the newest to the oldest.

The study by *Rigato, Johnson, Faraguna and Farroni, (2011)* involved two experiments (33.75% of the participants in the current study came from this study):

In the first two experiments (1a and 1b), the visual habituation procedure was used to investigate whether gaze direction modulates identity recognition from birth. Experiment 1a aimed at testing whether eye contact attracts newborns' attention and induces identity recognition from birth. Newborns were shown a face with direct gaze, and subsequently given a preference test involving the same face and a novel one, both of them with direct gaze. In Experiment 1b, the same procedure and face stimuli were used, in order to test identity recognition when faces displayed an averted gaze. Therefore, newborns were presented with a face accompanied with averted gaze, and subsequently given a preference test involving the same face and a novel one, both of them with averted gaze (Rigato et al., 2011).

In Experiment 2a and 2b, a visual preference paradigm was followed using the same stimuli (as in experiments 1a and 1b) accompanied with direct and averted gaze, respectively. The aim was to test whether newborns show a visual preference for one of the two faces. The apparatus was the same used in Experiment 1. The stimuli were the same face identities used in the previous experiments, and faces displayed direct gaze in Experiment 2a and averted gaze in Experiment 2b. The stimuli remained on the screen as long as the infants fixated one of them. When they shifted their gaze from the display for more than 10 s, the experimenter turned off the stimuli (Rigato et al., 2011).

The study by *Farroni, Menon, Rigato and Johnson, (2007)* involved three experiments (8.75% of the participants in the current study came from this study):

Experiment 1 aimed to establish if newborns have a spontaneous preference for fearful versus neutral facial expressions. Newborns were shown two pictures of the same person's face, one on the right and one on the left of the centre of the screen. One of the faces had a neutral face (no emotion) and the other had a fearful expression; both faces had a straight head and direct gaze. Two different identity faces were used, but each newborn saw only one of them (randomly assigned; Farroni et al., 2007).

Experiment 2 aimed to determine whether newborns could discriminate between the two stimuli

presented in Experiment 1 using a visual habituation and discrimination method. The apparatus and the stimuli were the same as these used in Experiment 1. During the habituation phase the newborns viewed pairs of identical face stimuli (same identity, same expression, one on the right and one on the left of the screen) with either a neutral expression or a fearful expression. During the test phase the two different expressions of the same identity face were presented bilaterally (Farroni et al., 2007).

Experiment 3 aimed to determine whether newborns could discriminate between stimuli with fearful expression and stimuli with happy facial expression. The apparatus was the same as that used in Experiment 1. Newborns were shown two pictures of the same person's face, one on the right and one on the left of the centre of the screen. One of the faces had a happy expression and the other had a fearful expression, both with a straight head and direct gaze. The two stimuli were 15.5 cm in all experiments (Farroni et al., 2007).

The study by *Farroni, Menon and Johnson, (2006)* involved three experiments (21.25% of the participants in the current study came from this study):

Experiment 1 aimed to establish whether newborns prefer to look significantly more at a face with direct gaze than at one with averted gaze. Newborns were shown two pictures of the same face: one on the right and one on the left of the centre of the screen. One of the faces had direct gaze, whereas in the other face the eyes were averted randomly to the right or left. The inner edges of the images were 8.5 cm from the centre (Farroni, Menon and Johnson, 2006).

Experiment 2 aimed to establish whether inverting faces affects gaze perception in newborns. The apparatus was the same as in Experiment 1 only this time the faces were inverted, with one face having direct gaze and the other face having a left or right averted gaze. The faces were presented at life size and 8.5 cm apart (Farroni, et al., 2006).

Experiment 3 followed procedures similar to those in Experiments 1 and 2 except that the faces used as stimuli were upright faces with the heads turned 45° to the left or right. As before, preferences for direct and averted gaze were compared. The faces were presented at life size and 8.5 cm apart (Farroni et al., 2006).

The study by *Farroni, Johnson, Menon, Zulian, Faraguna and Csibra, (2005)* involved two experiments (22.5% of the participants in the current study came from this study):

Experiment 1 included three parts (1a, 1b, 1c) and aimed to establish whether newborn's preference for upright schematic face-like configurations would disappear if they were composed of light elements on a dark background. The stimuli that were used in Experiment 1a were two head-shaped, head-sized, dimensional images with three-square features inside. One of the stimuli had the squares in the appropriate locations for eyes and mouth (i.e., an upright face-like configuration), whereas in the other stimulus the position of the squares was vertically reversed, with two squares located below one square (i.e., an inverted face-like configuration). In Experiment 1a the stimuli presented were differed only in contrast polarity: in the positive polarity condition the head-shape was white against a black background and the internal squares were black; in the negative polarity condition the head-shape was black against a white background and the internal squares were white. Experiment 1b used the same stimuli as in the negative polarity condition of Experiment 1a with the exception that the stimuli appeared on a mid grey (50 %) background. In Experiment 1c the stimuli used in Experiment 1b were changed slightly by inserting small black squares into the white ones (Farroni et al., 2005).

Experiment 2 included two parts (2a and 2b) and aimed to establish whether the contrast polarity sensitivity of newborns' preference is extended to real faces and across different lighting conditions. In Experiment 2a newborns were presented with two high-quality black-and-white photographs of a woman's face digitally modified to create an upright and an inverted version of it. The two stimuli were identical except for the inner region of the face, which was preserved in its canonical orientation in the upright face, but was rotated by 180° in the inverted face. Experiment 2a essentially replicated the findings of Experiment 1a. Experiment 2b tested directly whether newborns could discriminate between, and are biased towards one of, two faces, which are illuminated either from above or from below. The newborns were shown the same female face photographed with two different directions of illumination: from above and from below. The average luminance of the two stimuli was the same, while the distribution of the darker and lighter patches was markedly different. The face showed a neutral expression (Farroni et al., 2005).

Finally, eleven participants (13.75% of the participants) came from the following unpublished study: Di Gangi, V., Menon, E., De Pangher Manzini, E., and Farroni, T. (2008). La Costruzione dell'intenzionalità: uno studio sui neonati. Presentation at the XXII. Congresso Nazionale della Sezione di Psicologia dello Sviluppo. The study included two experiments (1: biological agent; 2: not biological agent) and aimed to evaluate whether there is evidence of goal-directed actions from birth. The stimuli were static scenes representing an agent touching one of two possible objects (two yellow bowling pins marked by a central red shape: a cross or a triangle). The participants were 31 full term newborns (20 for Exp. 1 and 11 for Exp.2).

Figures

Supplementary Figure S1. Scatter Plot showing the relationship between newborns' normalised average dwell time and normalised effortful control in childhood.

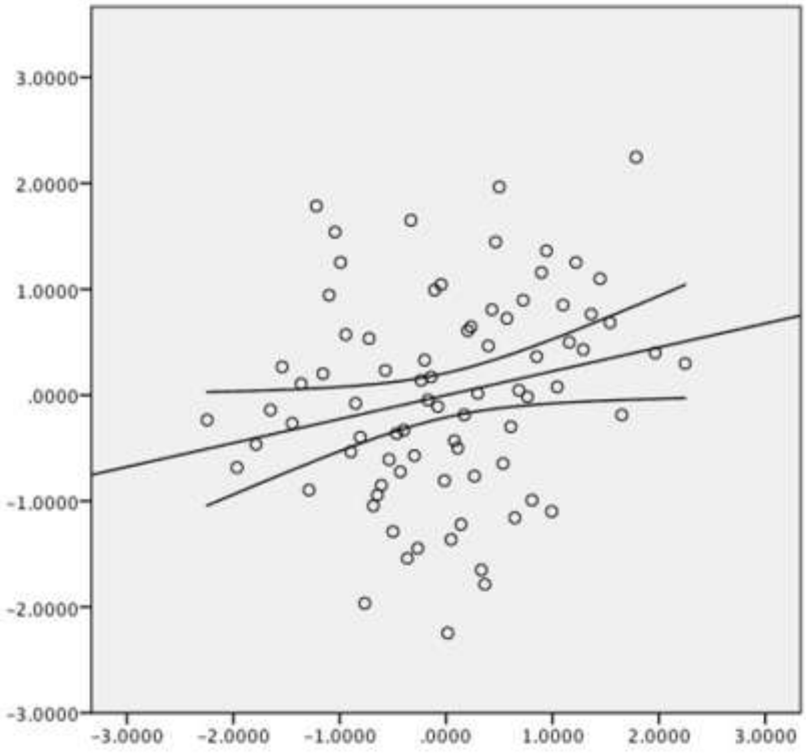

Note. The line represents the best-fit line of the model and the confidence bands surrounding the line represent the 95% confidence intervals of the best-fit line

Supplementary Figure S2. Scatter Plot showing the relationship between newborns' normalised average dwell time and normalised surgency in childhood.

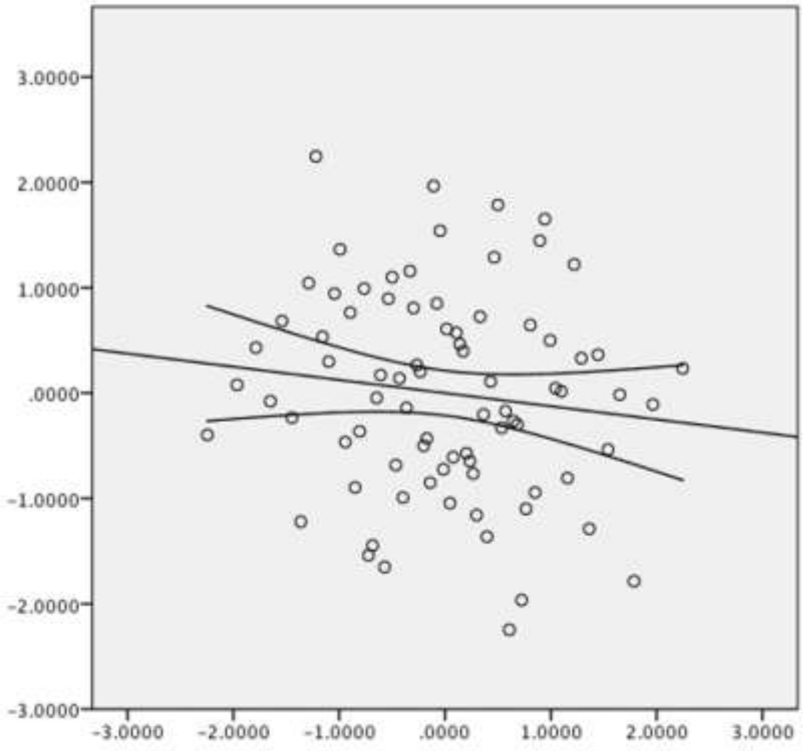

Note. The line represents the best-fit line of the model and the confidence bands surrounding the line represent the 95% confidence intervals of the best-fit line

Supplementary Figure S3. Scatter Plot showing the relationship between newborns' normalised average dwell time and normalised total behavioural difficulties in childhood.

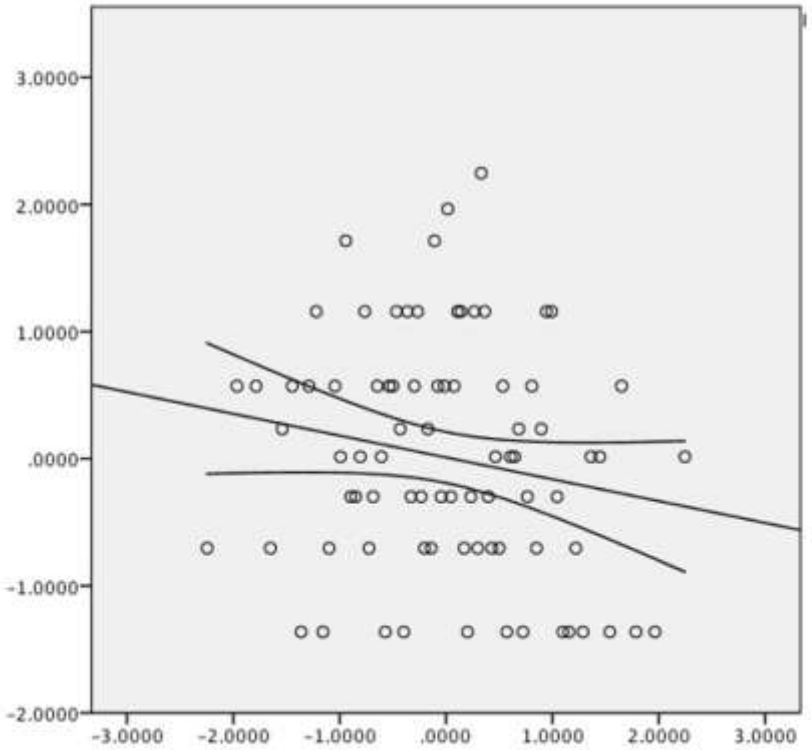

Note. The line represents the best-fit line of the model and the confidence bands surrounding the line represent the 95% confidence intervals of the best-fit line

Tables

Supplementary Table S1. Descriptive Statistics for CBQ and TMCQ scales of Effortful Control and Surgency and the

	CBQ (5-7 years of age)		TMCQ (7-9 years of age)		SDQ (5-7 years of age)	SDQ (7-9 years of age)
	Effortful Control	Surgency	Effortful Control	Surgency	SDQ Total Behavioral Difficulties Score	SDQ Total Behavioral Difficulties Score
N	13	13	67	67	13	67
Mean	5.69	5.01	3.47	3.45	6.53	7.80
SD	.44	1.02	.47	.42	4.27	5.66
Median	5.65	5.07	3.46	3.45	6.00	7.00
Mode	4.99	2.71	3.47	2.60	3.00	2.00
Minimum	4.99	2.71	2.17	2.60	.00	0.00
Maximum	6.59	6.59	4.57	4.50	15	27.00
Kurtosis	.27	1.18	-.15	.17	-.29	1.47
Skewness	.52	-.75	-.12	.31	.55	1.09
Cronbach's Alpha	.75	.91	.90	.89	.76	.83

SDQ scale of Behavioural Difficulties

Supplementary Table S2. Mean Sex Differences on Average Dwell Time and Effortful Control, Surgency and Total Behavioral Difficulties

N=80 (5-9 years of age)	Male (N = 44)		Female (N = 36)					
	M	SD	M	SD	F	df	p-value	Partial Eta-squared
Average Dwell Time	3,583	1,592	4,083	2,333	.04	1, 61	.84	.001
Effortful Control	-.24	.87	.30	1.06	5.92	1, 76	.01	.05
Surgency	.07	1.02	-.09	.95	.57	1, 76	.45	.008
Total Behavioral Difficulties	7.50	4.92	7.72	6.12	.03	1, 76	.86	.000

Supplementary Table S3. Mean Group Differences on Total Looking Time at Stimuli, Total Number of Orientations and Average Dwell Time between participants that took part in Visual Preferences and Visual Habituation Studies

N=80 (6-9 years of age)	Preferences Studies (N = 56)		Habituation Studies (N = 24)					
	M	SD	M	SD	F	df	p-value	Partial Eta-squared
Total Looking Time at Stimuli	97,465	3.81	49,203	1.58	41.92	1, 79	.000	.36
Total Number of Orientations	29.44	11.19	15.19	6.13	35.46	1, 79	.000	.31
Average Dwell Time	3,735	1,651	3,960	2,523	.005	1, 79	.94	.000
